# Editorial: Cell death programs in the pathogenesis of heart disease

**DOI:** 10.3389/fphys.2025.1581282

**Published:** 2025-03-14

**Authors:** Jennifer Q. Kwong, Robert N. Correll, Qinghang Liu

**Affiliations:** ^1^ Department of Cell Biology, Emory University, Atlanta, GA, United States; ^2^ Department of Biological Sciences, University of Alabama, Tuscaloosa, AL, United States; ^3^ Center for Convergent Bioscience and Medicine, University of Alabama, Tuscaloosa, AL, United States; ^4^ Department of Neurobiology and Biophysics, University of Washington, Seattle, WA, United States

**Keywords:** cell death, heart disease, apoptosis, ferroptosis, pyroptosis

Cell death plays an important role in the pathogenesis of multiple forms of heart disease such as myocardial infarction, myocarditis, cardiomyopathies, drug-induced cardiotoxicity, and heart failure of diverse etiologies. Apoptosis is the first identified form of regulated cell death, which is characterized by cell shrinkage, membrane blebbing, chromatin condensation, and DNA fragmentation, without loss of plasma membrane integrity or inflammatory response (Danial and Korsmeyer, 2004). In contrast, necrosis had long been regarded as an unregulated process characterized by loss of plasma membrane integrity, cell swelling and lysis, and marked tissue inflammation and fibrosis (Edinger and Thompson, 2004). However, recent studies have identified multiple regulated necrosis programs, including necroptosis, ferroptosis, pyroptosis, parthanatos, mitochondria-mediated necrosis, and other regulated necrotic processes (Del Re et al., 2019). The discovery of regulated necrosis opens a new avenue to target necrosis for the treatment of heart disease, which was deemed impossible in the past. These cell death programs are often activated by distinct extracellular or intracellular signals under different physiological or pathological settings. The goal of this review series is to provide an overview of recent findings pertaining to the role of various cell death programs in the pathogenesis of heart diseases (Grisanti; Bhatti and Sato; Rowland et al.; Fatima et al.).

Apoptosis can be engaged through two primary pathways: the mitochondria-dependent intrinsic pathway or the extrinsic pathway which involves ligand-mediated activation of death receptors on the plasma membrane. While research on cardiac apoptotic death mechanisms have primarily focused on delineating the molecular effectors of intrinsic apoptosis as well as extrinsic apoptosis mediated by the pro-inflammatory cytokines like tumor necrosis factor (TNF)-α in the heart, Grisanti sheds light on the emerging roles of TRAIL (TNF-related apoptosis-inducing ligand), a member of the TNF superfamily known for its role in cancer cell apoptosis, but is less studied in the context of the heart. TRAIL and its receptors, particularly DR5 (death receptor 5), have been associated with key cardiovascular disease risk factors including smoking, diabetes, and hypertension, and as well as cardiac diseases including arrhythmias, atherosclerosis, acute myocardial infarction, and heart failure. Notably, the relationship between TRAIL and cardiovascular disease may be complex, as human studies suggest both pro-apoptotic and cardioprotective effects. This duality underscores the importance of defining TRAIL’s roles in different cardiac cell types and pathologies, which could pave the way for future development of TRAIL-targeted therapies.

Beyond apoptosis, this Research Topic also highlights two emerging forms of regulated necrosis that can also contribute to cardiac disease: ferroptosis (Fatima et al.) and pyroptosis (Bhatti and Sato). Ferroptosis is a form of regulated cell death characterized by iron-dependent lipid peroxidation. Intracellular iron overload, which can be triggered by dysregulated autophagy of ferritin (ferritinophagy) or aberrant heme degradation, induces lipid peroxidation via the Fenton reaction and cytotoxic damage. Moreover, inactivation of key suppressors of lipid peroxidation, including glutathione peroxidase 4 (GPX4) and ferroptosis suppressor protein-1 (FSP-1), can also induce ferroptotic death. Pyroptosis on the other hand, is a form of regulated necrosis associated that is closely associated with inflammation. In this form of death, activation of inflammation-associated caspases through canonical (caspase-1 dependent) or non-canonical (caspase-4/-5/-11 dependent) pathways lead cleavage and activation of pore-forming gasdermin proteins like GSDMD, which can permeabilize the plasma membrane leading to the release of pro-inflammatory signals. While both ferroptosis and pyroptosis have independently been implicated in a host of cardiac pathologies, notably, they both contribute to myocardial infarction/reperfusion injury, doxorubicin-induced cardiomyopathy, and hypertrophic cardiomyopathy, suggesting that these pathways may interact. Indeed, Fatima et al. discuss the highlight the crosstalk between ferroptosis and pyroptosis, as deletion of GPX4, a central regulator of ferroptosis, can engage pyroptosis through the activation of caspase 11, leading to the cleavage and activation of GSDMD. This potential crosstalk between ferroptosis and pyroptosis suggests a complex interplay of death pathways in cardiac disease and future work aimed at delineating how different death mechanisms intersect and defining key regulatory nodes may hold the key to limiting cardiomyocyte cell death in cardiovascular disease.

Cell death pathways in supporting cells of the heart also play important roles in disease pathogenesis. For example, fibroblasts are involved not only in maintenance of the cardiac extracellular matrix (ECM) but are also activated in response to stress and injury. Myocardial infarction results in fibroblast differentiation into myofibroblasts (Ivey et al., 2018) that proliferate to provide the heart with mechanical support and prevent rupture (Davis and Molkentin, 2014). Hypertrophic remodeling and subsequent decompensation are also associated with the development of fibrosis (Ivey et al., 2018; Xia et al., 2009). This places cardiac fibroblasts in an environmental context that may activate unfolded protein response (UPR) signaling that can initiate cell death pathways under prolonged stress (Rowland et al.). However cardiac fibroblasts also demonstrate resistance to apoptosis and fibroblast overactivation is a major contributor to adverse cardiac remodeling and heart failure progression (Meng et al., 2018). Thus, future studies investigating how cardiac fibroblasts evade cell death may suggest strategies to limit pathological remodeling and disease progression.

Recent studies clearly demonstrate that multiple cell death programs, including apoptosis, necroptosis, ferroptosis, pyroptosis, and other cell death modalities, contribute to the pathogenesis of heart diseases. Genetic or pharmacologic inhibition of these cell death programs showed beneficial effects in experimental models of heart disease such as myocardial infarction and heart failure. Therefore, targeting cell death represents a promising therapeutic strategy for various forms of heart disease, although these approaches warrant further investigation in clinical settings. Of note, accumulating evidence suggests that besides cardiomyocytes, cell death in other cell types, including endothelial cells, smooth muscle cells and fibroblasts, also contributes the pathogenesis of heart disease. Moreover, the relative contributions of various cell death programs under specific disease conditions remain unclear, partly due to the lack of reliable and specific cell death markers and other diagnostic tools, which warrants further investigation. The crosstalk between various cell death mechanisms further increases the complexity of these cell death pathways in disease pathogenesis. Recent studies suggest that simultaneous targeting multiple cell death programs may serve as a valid strategy for the treatment of heart disease.

## References

[B1] Danial N. N. Korsmeyer S. J. (2004). Cell death: critical control points. Cell 116, 205–219. 10.1016/S0092-8674(04)00046-7 14744432

[B2] Davis J. Molkentin J. D. (2014). Myofibroblasts: trust your heart and let fate decide. J. Mol. Cell. Cardiol. 70, 9–18. 10.1016/j.yjmcc.2013.10.019 24189039 PMC3995855

[B3] Del Re D. P. Amgalan D. Linkermann A. Liu Q. Kitsis R. N. (2019). Fundamental mechanisms of regulated cell death and implications for heart disease. Physiol. Rev. 99, 1765–1817. 10.1152/physrev.00022.2018 31364924 PMC6890986

[B4] Edinger A. L. Thompson C. B. (2004). Death by design: apoptosis, necrosis and autophagy. Curr. Opin. Cell Biol. 16, 663–669. 10.1016/j.ceb.2004.09.011 15530778

[B6] Ivey M. J. Kuwabara J. T. Pai J. T. Moore R. E. Sun Z. Tallquist M. D. (2018). Resident fibroblast expansion during cardiac growth and remodeling. J. Mol. Cell Cardiol. 114, 161–174. 10.1016/j.yjmcc.2017.11.012 29158033 PMC5831691

[B7] Meng Q. Bhandary B. Bhuiyan M. S. James J. Osinska H. Valiente-Alandi I. (2018). Myofibroblast-specific TGFβ receptor II signaling in the fibrotic response to cardiac myosin binding protein C-induced cardiomyopathy. Circ. Res. 123, 1285–1297. 10.1161/CIRCRESAHA.118.313089 30566042 PMC6309316

[B8] Xia Y. Lee K. Li N. Corbett D. Mendoza L. Frangogiannis N. G. (2009). Characterization of the inflammatory and fibrotic response in a mouse model of cardiac pressure overload. Histochem. Cell. Biol. 131, 471–481. 10.1007/s00418-008-0541-5 19030868 PMC2782393

